# Detection and Recognition of Voice Commands by a Distributed Acoustic Sensor Based on Phase-Sensitive OTDR in the Smart Home Concept

**DOI:** 10.3390/s24072281

**Published:** 2024-04-03

**Authors:** Tatyana V. Gritsenko, Maria V. Orlova, Andrey A. Zhirnov, Yuri A. Konstantinov, Artem T. Turov, Fedor L. Barkov, Roman I. Khan, Kirill I. Koshelev, Cesare Svelto, Alexey B. Pnev

**Affiliations:** 1 Laser and Optoelectronic Systems Department, Radio Electronics and Laser Technology Faculty, Bauman Moscow State Technical University, 2-nd Baumanskaya 5-1, 105005 Moscow, Russia; chobantv@yandex.ru (T.V.G.); manja254@yandex.ru (M.V.O.); a.zh@bmstu.ru (A.A.Z.); khan.roman.igorevich@gmail.com (R.I.K.); koshelev-k@yandex.ru (K.I.K.); 2 Perm Federal Research Center of the Ural Branch of the Russian Academy of Sciences (PFRC UB RAS), 13a Lenina St., 614990 Perm, Russia; yuri.al.konstantinov@ro.ru (Y.A.K.); artemtur442@gmail.com (A.T.T.); barkov.f@permsc.ru (F.L.B.); 3 General Physics Department, Applied Mathematics and Mechanics Faculty, Perm National Research, Polytechnic University, Prospekt Komsomolsky 29, 614990 Perm, Russia; 4 Dipartimento di Elettronica, Informazione e Bioingegneria Politecnico di Milano, Piazza Leonardo da Vinci 32, 20133 Milano, Italy; cesare.svelto@polimi.it

**Keywords:** fiber-optic sensor, distributed fiber-optic sensor, phi-OTDR, acoustic monitoring, machine learning, speech recognition

## Abstract

In recent years, attention to the realization of a distributed fiber-optic microphone for the detection and recognition of the human voice has increased, whereby the most popular schemes are based on φ-OTDR. Many issues related to the selection of optimal system parameters and the recognition of registered signals, however, are still unresolved. In this research, we conducted theoretical studies of these issues based on the φ-OTDR mathematical model and verified them with experiments. We designed an algorithm for fiber sensor signal processing, applied a testing kit, and designed a method for the quantitative evaluation of our obtained results. We also proposed a new setup model for lab tests of φ-OTDR single coordinate sensors, which allows for the quick variation of their parameters. As a result, it was possible to define requirements for the best quality of speech recognition; estimation using the percentage of recognized words yielded a value of 96.3%, and estimation with Levenshtein distance provided a value of 15.

## 1. Introduction

Smart homes, smart manufacturing, and even smart cities are increasingly becoming part of the lives of citizens in many countries worldwide [1,2,3]. Some use these technologies as luxury items, but for people with disabilities, these are a few ways to improve the quality of one’s life to an acceptable level. In smart manufacturing, artificial intelligence and Internet of Things (IoT) technologies can be integrated into one sensor system as a way of organizing rational and comfortable work. In large cities, such technologies allow for monitoring and regulating passenger and human traffic, structures, personal identification, etc. [4,5]. The key feature of the sensors in such intelligent systems is their ability to acquire information without distraction from daily activities. One of the most common and simplest control methods from the point of view of human physiology is a voice command [6,7]. However, using an example of a smart home, we can easily see that not all IoT devices have voice input devices (microphones)—for example, refrigerators and microwave ovens are typically not controlled by voice. The same applies to technological and laboratory equipment in smart production. Even if we imagine that all pre-machine or factory peripherals are equipped with voice input devices, many new questions arise: what frequency and bitrate are necessary to digitize a voice signal? How should we transmit it to the network—in the form of an original audio file or text strings? In any case, adding a microphone, an analog-to-digital converter, and a data transmission channel to each element of a smart system would require significant costs, and the integration of new electronic components would also require additional electrical power. In this paper, we propose a method for registering voice commands using a distributed fiber-optic sensor. Distributed optical fiber sensing technologies have become widespread nowadays [8,9,10]. Rayleigh scattering sensors, which operate on the principle of phase-sensitive optical reflectometry in the time domain, are the most sensitive to acoustic frequency fluctuations. The sensitive element in such sensors is the optical fiber itself, which enables the measurement of various physical parameters of the environment [11,12,13], the separate registration of acoustic signals and disturbances in different parts of extended objects and locations along the entire length of the sensing fiber, and the accurate determination of disturbance coordinates. In 1977 [14], it was shown that acoustic disturbances affect parameters of light propagation in sensing fibers, demonstrating the possibility of creating a fiber-optic microphone; today, such devices are highly interesting to potential users. Fiber-optic microphones have the following significant advantages over common electronic microphones: they are compact and light, which allow them to be placed in hard-to-reach places; they can record audio information without electromagnetic interference; they can be placed in fire-safe zones and conditions of high and low temperatures; and there is no need for electric wiring and local power supplies. A fiber microphone can be included in an existing fiber communication line for recording information and for its direct transmission, which makes fiber-optic microphones promising audio devices for use in communication systems as well.

**Figure 1:**
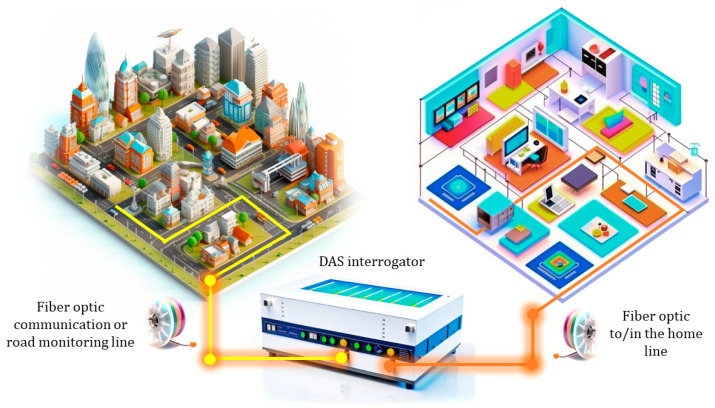
Scheme of DAS interrogation for a smart city (**left**) and smart home (**right**).

The advantages of fiber-optic microphones have attracted the attention of researchers. Various schemes for their construction have been proposed, including those based on fiber interferometers, including weak Fiber Bragg Gratings (wFBGs), and those based on phase-sensitive optical reflectometers in the time domain (φ-OTDR), which are commonly used as distributed acoustic sensors (DAS) [15]. A distributed fiber-optic microphone based on φ-OTDR is a promising technology that allows for the recording of acoustic disturbances, including speech, along a sensing fiber with a high resolution of 10 m [16] and in a wide frequency band [10] all over large objects and locations with lengths of up to 80 km. A distributed fiber microphone based on φ-OTDR can be used as an independent system to obtain information about speech signals and their coordinates. In addition, it can significantly expand the functions of security systems and monitoring systems for extended objects, such as pipelines, railways, and long-perimeter objects, to prevent and detect leaks and unauthorized tie-ins and perimeter crossings, as well as be integrated into smart home and smart city technologies. The basic concept of projecting the approach of DAS-equipped smart cities onto the technology of a DAS-equipped smart home is shown in Figure 1.

In recent years, various research groups have been working to create a distributed fiber microphone for recording human voice based on a φ-OTDR [12,13,17]. It was demonstrated that a φ-OTDR can be used to record single-frequency sound disturbances by speakers at specified frequencies in a range of 2–3 kHz, which can be subsequently restored from the received signals and reproduced [11]. In [12], the English alphabet dictated through a loudspeaker was recorded using a φ-OTDR-based microphone, and in [13,17], the authors recorded human speech amplified through a loudspeaker adjusted to the C-weighted sound pressure level (dB(C)) of 85 dB using such a microphone. All these studies demonstrate the significant potential of this technology. However, many problems have not been solved to date. The most important parameter of a distributed fiber-optic microphone is the quality of speech signal recognition. It was noted in [17] that, at the moment, researchers use subjective criteria (e.g., [18]) to determine this indicator. It seems necessary to characterize the recognition quality quantitatively and objectively. To solve this problem, we propose using open speech recognition services.

The issue of selecting the optimal parameters of the φ-OTDR scheme to ensure the highest quality of speech registration is also relevant to enabling the further reproduction and recognition of words. Most of this study is also devoted to this issue. We studied the influence of φ-OTDR parameters—such as the pulse repetition rate and the required amplification of radiation directed into the line (and then the one backscattered)—on the quality of human speech recording and recognition. A study of the quality of human speech recording and recognition dependence on parameters of an φ-OTDR in the form of pulse repetition rate and sound volume was conducted. Separately, we would like to highlight that the authors considered the issue of voice recognition using a phase-sensitive reflectometer with direct (incoherent) detection without phase recovery. Thus, the method used has a nonlinear transfer characteristic toward external disturbances, which somehow leads to lower-quality signal transmission and recognition results but allows using a much simpler φ-OTDR scheme for detection. In addition, this technique is less susceptible to outliers that appear during the phase-reconstruction procedure at moments of too-rapid linear growth in a noisy urban environment. In any case, the newly proposed quantitative and objective method for assessing the quality of signal recognition applies to any type of fiber-optic sensor. The results of phase recovery in the case of a linear transfer characteristic during our experiments can be observed when analyzing the signal from a pair of wFBGs, which form a sensitive section similar in principle to the survey section of the reflectometer in terms of phase recovery. Devices like φ-OTDR without phase recovery are easy to assemble and stable in operation; therefore, their use as a distributed microphone is important and promising for widespread application.

## 2. Theory

### 2.1. The φ-OTDR Operation Principle

The φ-OTDR can provide information about the presence of external acoustic and vibration disturbances on the fiber sensor throughout its entire length in real time. It ensures localization and speech recognition at any point along the sensing fiber. Thus, one can, over time, monitor and receive information about which voice command is given and at which location of the apartment or the room, or recognize the speech of several speakers separately at once.

**Figure 2:**
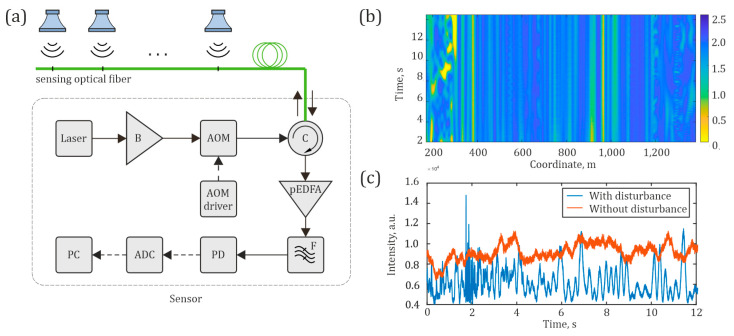
(**a**) Scheme of a distributed fiber microphone based on a φ-OTDR; (**b**) waterfall of backscattered intensity (in a fake color scale) as a function of time and coordinate; and (**c**) backscattered intensity as a function of time for a specific coordinate.

The scheme of a distributed fiber microphone based on a φ-OTDR is presented in Figure 2. The operation principle of a distributed fiber-optic microphone based on a φ-OTDR is as follows: Radiation from a laser source, with a coherence time greater than the adopted pulse width (typically in the order of 100 ns), propagates throughout the scheme. Firstly, the laser output passes through an erbium-doped fiber amplifier (EDFA) booster (B), which increases the radiation power to the required level. Next, an acousto-optic modulator (AOM) generates light pulses sent through the circulator (C) in the sensing fiber. The sensing fiber is located in a cable, which is mounted along a monitored object or space. The modulated light propagates along the fiber with low attenuation. It is scattered back because of the refractive index inhomogeneities in the glass, which appear during the fiber production process or arise/change in the presence of external disturbances. From each section of the fiber, with a spatial resolution *l*_0_ determined by the half-width duration of the optical pulse at the output of the AOM, backscattered lightwaves interfere with one another, producing high- and low-intensity peaks. Through C, the returning (backscattered) interference optical signal travels to a preamplifier (pEDFA), which amplifies it to increase the signal level adequately for high-precision optical detection. After the preamplifier, the pEDFA broadband spontaneous radiation (amplified spontaneous emission (ASE), noise) is filtered out from the signal using a spectrally selective narrow-bandpass optical filter (F) centered at the laser wavelength, and the signal is finally sent to a photodetector (PD). The voltage output from the PD is digitized by an ADC. As a result of the interference of backscattered lightwaves with random phases, a rugged reflectogram is formed, providing the dependence of recorded signal intensity on time for each point (or coordinate) along the fiber. A sequence of reflectograms, each retrieved from different backscattered light pulses, is called a “waterfall”. An example of a “waterfall” reflectogram is shown in Figure 2b. It is a three-dimensional representation of the signal, which shows an array of all the reflectograms, depending on the spatial coordinates, with their changes over time, showing intensity variations between different pulses (temporal evolution) and coordinates (spatial evolution) along the sensing fiber. The “waterfall” cross-section, which is the backscattered intensity as a function of time for a specific coordinate, is presented in Figure 2c.

In the absence of external acoustic and vibration disturbances acting on the optical fiber, the reflectogram has a more stable appearance, influenced only by some electrical and thermal noise. In the presence of an acoustic disturbance, the scattering centers in the fiber are shifted relative to their initial position, and new scattering centers are induced due to the deformation of the fiber and concomitant changes in the refractive index. As a result, the reflectogram changes significantly over time, also depending on the amplitudes and frequencies of the vibration disturbance (as well as a voice signal). After receiving the sequence of reflectograms over time, it is possible to form a “waterfall”, as shown in Figure 2b. Its analysis shows quiet and influenced sensor sections and can be used to recognize the disturbance type and its parameters. The point signal in the form of an intensity evolution in time, an example of which is shown in Figure 2c, is preprocessed at each moment of time. After accumulation over some characteristic duration (usually a few seconds), voice signals can be recognized and converted into text.

### 2.2. Mathematical Model of the Signal-Formation Process in a Distributed Fiber-Optic Microphone Based on φ-OTDR

For a theoretical study of the quality of speech recognition dependence on such φ-OTDR scheme parameters, a mathematical model was developed by forming a signal from a single point of a distributed fiber-optic microphone based on φ-OTDR.

The signal recorded by the ADC at each moment of time can be represented as a dependence of the intensity of the interference pattern on time *I*(*t*):

(1) $$I\left(t\right)={\left|\sum_{i=1}^{N}A_{n}\cdot e^{j\varphi_{n,t}}\right|}^{2}$$

where *N* is the number of scattering centers distributed randomly over a 20 m fiber length section (corresponding to the optical pulse half-duration of 100 ns); *A_n_* is the amplitude of the lightwave reflected from the *n*-th scattering center, which is constant over time; and *φ_n,t_* is the phase of the lightwave reflected from the *n*-th scattering center.

Since the φ-OTDR uses a narrow-band radiation source, for which the coherence length is much longer than the pulse width along the fiber, the sum of backscattered lightwaves from one pulse takes into account the phases, as can be seen in Equation (1) (i.e., it is coherent within the pulse length). In this case, the amplitudes of the backscattered lightwaves obey a distribution that can be taken as normal (since we have a large number of randomly distributed scattering centers along the pulse within the fiber), and the phase distribution is uniform over an interval of [−π; π]. Thus, the probability distributions of the amplitude *p*(*a*) and phase *p_θ_*(*θ*) of the resulting signal from the scattering centers can be described by the following expressions:

(2) $$p\left(a\right)=\left\{\begin{array}{c} \frac{1}{{\sigma \sqrt{2\pi }}^{\;}}exp\left\{-\frac{1}{2}{\left(\frac{a_{0}-a}{\sigma }\right)}^{2}\right\}\\ 0\;\mathrm{otherwise} \end{array}\right.\;\mathrm{if}\;a>0$$

(3) $$p_{\theta }\left(\theta \right)=\left\{\begin{array}{c} \frac{1}{2\pi }\;\mathrm{for}-\pi <\theta \leq \pi \\ 0\;\mathrm{otherwise} \end{array}\right.$$

where *σ* is the amplitude standard deviation, *a*_0_ is the amplitude mean value, and *a* is the amplitude of a given scattering center.

The signal at the system’s output is noisy due to the instability of the system’s components’ parameters. The main sources of signal distortion are discussed below.

Firstly, laser wavelength fluctuations are a source of signal distortion. This affects both the system sensitivity and the transfer function linearity. These dependencies are due to the interferometric nature of the signal [19]. The interference leads to maxima and minima in the intensity changes in time, which is the interference pattern. The sensitivity of the system is greater in intensity peaks and minimal in low-intensity zones [20]. Further, random multibeam interference causes nonlinearity of the transfer function of the system, which differs from the two-beam interference simple sinewave, which is observed, e.g., when two waves are reflected from a pair of wFBGs. The transfer function differs for each sensing fiber coordinate, being defined by the random location of the scattering centers at each point as well as by backscattered wave amplitudes and phase distributions within the pulse. 

Secondly, signal distortion is caused by the noise of the receiver components, such as a photodiode with an electrical transimpedance amplifier and eventually a pEDFA. At long working distances, a pEDFA is absolutely necessary. In most cases, average PD noise remains quite the same, while a pEDFA with a large gain both helps to increase a signal level and introduces additional noise. When the pEDFA noise is filtered out, the resulting signal-to-noise ratio is increased. Thus, pEDFA noise can be considered the dominant term.

To simulate processes occurring in the fiber-optic microphone based on a φ-OTDR, the following mathematical model was created.

The laser has frequency instability over time, which can be described by the following expression:

(4) $$\nu =\nu_{0}+\left|F^{-1}\left\{\sqrt{S_{\nu }{f_{rep}}^{2}T}\right\}\right|,$$

where $\nu_{0}$ is an initial laser frequency in Hz, *F*^−1^ {…} is the inverse Fourier transform, and *S_ν_* = *Š_ν_*∙|*F*{rand(1)}|^2^ ∙ 1/*ν_p_*^2^. *T* is the simulated power spectral density of laser frequency oscillations with envelope *Š_ν_* during the observation time *T*, and *f*_rep_ is the pulse repetition rate.

To describe the phase, it is necessary to take into account the wavelength *λ_t_* of the radiation source and the distance *L_n_* from the circulator (or some other conditional point) to the scattering center. In a fiber, the average size of inhomogeneities on which Rayleigh scattering occurs is about 1/10 of the wavelength, so the number of scattering samples over the fiber length *L*_S_ is

(5) $$N_{p}≅\frac{L_{s}}{\lambda /10}$$

Therefore, for a sensor with a length of *L*_S_ = 20 m, the number of scattering centers at a wavelength of *λ* ≈ 1550 nm will be *N_p_* = 1.3 × 10^8^. The inhomogeneities are randomly located along the entire length of the fiber. Under such conditions, we can describe the distance from the circulator to the *n*-th scattering center as

(6) $$L_{n}=N_{p,n}\cdot \frac{\lambda }{10}\pm \frac{\lambda }{100}\cdot rand\left(1\right)$$

where *N_p,n_*∙*λ*/10 is the distance from the circulator to the *n*-th scattering center, and ±*λ*/100∙rand(1) is a random shift used to simulate random positions of the scattering centers to obtain the random phase distribution. External disturbance to the sensor leads to a shift of the scattering centers in the fiber and, consequently, to a change in the phase of the backscattered signal from each scattering center:

(7) $${∆\varphi }_{n,t}=\frac{4\pi n}{\lambda }{(L}_{n}+∆L_{n,t}),$$

where *n* is the effective group refraction index and ∆*L_n,t_* is a displacement of the scattering center coordinate in the presence of an external disturbance:

(8) $$∆L_{n,t}={\Delta L}_{max}\cdot \frac{l}{l_{max}}\cdot D(t),\;0\leq l\leq l_{max}$$

where Δ*L*_max_ is the amplitude of the disturbance (in terms of scattering center displacement), *l*_max_ = 20 m is the length of the fiber being tested, and *D*(*t*) is the normalized shape of the disturbance applied to the fiber sensor. Here, we assumed that the sensing fiber is wound on a piezoelectric transducer (PZT), as it is a realistic and simplest case of signal emulation [21]. 

The given distributions and dependencies allowed us to numerically simulate a noisy signal, including the wavelength instability of the laser source. The second main part of the noise is either the noise of the photodetector or the noise of the preamplifier. The noise of the preamplifier is of the greatest interest since a pEDFA is used in most φ-OTDR schemes. For pEDFA noise calculation, the power of the scattered radiation in a 20 m coil of PZT can be calculated as follows. The laser input power is taken as *P*_in_ = 10 dBm. Taking into account the losses of connectors and the system components (such as the circulator), and using dBm and dB values, we can obtain

(9) $$P_{out}=P_{sig}+G$$

(10) $$P_{sig}=P_{in}+\eta +K_{att}+r$$

where *r* = *r*_1_ + 10log(*τ*) is a Rayleigh scattering coefficient, *τ* is a pulse width (FWHM), *η* = −4 dB is the loss of components, and *K*_att_ = −2*αL* ≈ 0 is the attenuation in *L* = 20 m of fiber with an attenuation coefficient of *α* = 0.2 dB/km. For standard single-mode fiber, *r*_1_ = −80 dB is the part of radiation scattered by a pulse with a width *τ* = 1 ns. For a spatial resolution of *l*_0_ = 20 m, the corresponding pulse width is *τ* = 200 ns, so *r* = −57 dB. Preamplifier gain for a low-power signal is about *G* = 47 dB. In such conditions, we obtain a scattered power of *P*_sig_ = 7.9 nW, which is amplified by the pEDFA to *P*_out_ = 0.4 mW.

The noise of the preamplifier can be divided into spontaneous–spontaneous and signal–spontaneous [22]. The dispersion (variance) of the beat noise of the signal with spontaneous emission in the optical preamplifier is

(11) $${\sigma_{s-sp}}^{2}=2G^{2}P_{sig}NFh\nu ∆f=5.64\cdot 10^{-9}\;W^{2}$$

where *G* = 50,000 (47 dB) is the preamplifier gain coefficient, *NF* = 4 is the preamplifier noise figure, *h* = 6.626∙10^−34^ J∙s is the Planck constant, *ν* = 193.4 THz is the radiation frequency (*λ* = 1550.12 nm), and ∆*f* = 200 MHz is the photodetector bandwidth.

The beating noise of spontaneous with spontaneous radiation in the preamplifier is

(12) $${\sigma_{sp-sp}}^{2}={\left(NF\cdot h\cdot \nu \cdot G\right)}^{2}\cdot 2\cdot ∆f\left(∆\nu -0.5\cdot ∆f\right)=3.27\cdot 10^{-10}\;W^{2}$$

where ∆ν=1 GHz is the optical filter bandwidth, which determines the quantity of passed spontaneous emission noise. Using such parameter values, and in particular an ASE noise optical filter as narrow as 1 GHz (∆*λ* = 8 pm), we see that the spontaneous–spontaneous noise is negligible (<1/10) with respect to signal–spontaneous noise.

The signal intensity after the pEDFA can be described as follows:

(13) $$I_{g}\left(t\right)=G\cdot I\left(t\right)+N_{ind,t}$$

where $N_{ind,t}=\sqrt{\sigma_{s-sp}^{2}+\sigma_{sp-sp}^{2}}≅\sigma_{s-sp}^{2}$ is the total preamplifier noise.

### 2.3. Principles of the Signal-Formation Process in a Distributed Fiber-Optic Microphone Based on φ-OTDR with wFBGs 

The Rayleigh backscatter signal generated by natural refractive index inhomogeneities in a common sensing fiber has a naturally low intensity, which results in a low signal-to-noise ratio in the output of a microphone based on a φ-OTDR. To increase the scattering level [23,24], which improves the quality of speech recognition, a fiber with wFBGs can be used [25]. The back-reflected signal power fraction arriving at the PD for a wFBG with a reflection coefficient of 0.1% is 10^−3^, while for Rayleigh scattering in a common fiber, the power fraction is 10^−6^, 3 orders of magnitude weaker.

In the case when a microphone is based on Rayleigh scattering from refraction index inhomogeneities, the output signal is an interference of the backscattered lightwaves within the half-width of a pulse. A phase change of each wave due to a scattering inhomogeneity displacement makes a nonlinear contribution to the final interference signal changes. When a section of the fiber is stretched, the change in the interference signal is determined both by the amplitude of the disturbance and a phase, depending on the distance to the scattering center within the section. As a result, an interference signal from all the scattering centers does not simply change sinusoidally but rather according to a complex harmonic law. When there are specially applied artificial reflectors, such as wFBGs, in the fiber, the signal is an interference of only two waves reflected from neighboring wFBGs, taking into account a phase difference in these two waves. When an acoustic disturbance affects a section of the sensing fiber located between two wFBGs and stretches at the length Δ*l*, the phase difference of the waves reflected from the first and second gratings changes by the value of Δ*φ = n*Δ*l·2π/λ*, and the signal is mathematically expressed as a sum of two waves, according to (14). Thus, each pair of wFBGs in the fiber forms a sensitive section, which is similar to polling a section of a φ-OTDR with phase recovery.

(14) $$I=I_{1}+I_{2}+2\sqrt{I_{1}I_{2}}\cos\left(\left(\varphi_{2}+\Delta \varphi \right)-\varphi_{1}\right)\;$$

where $I_{1,2}$ is the intensity of the lightwave reflected from the first and the second grating, respectively, and $\varphi_{1,2}$ is the phase of the lightwave reflected from the first and the second grating, respectively.

### 2.4. Numerical Modeling of the Signal in a Distributed Fiber-Optic Microphone Based on φ-OTDR 

To verify the possibility of operating the φ-OTDR as a distributed microphone, numerical modeling was carried out according to the developed mathematical model. In order to conduct correct, repeatable, and representative studies, it is important to choose an adequate type of disturbance affecting the sensor. The modulation of the influencing signal by a sound wave of human speech containing Harvard sentences [26] was chosen as the test signal (disturbance). These sentences are phonetically balanced standardized phrases developed in 1965 to assess the transmission quality of speech signals over a communication line. Ten Harvard sentences were selected, so the following sequence presented in Listing 1, with a total duration of about 40 s and containing 80 words (*M =* 386), was used as a speech disturbance.
**Listing 1.** Harvard sentences used for speech recognition.The birch canoe slid on the smooth planks.Glue the sheet to the dark blue background.It’s easy to tell the depth of a well.These days a chicken leg is a rare dish.Rice is often served in round bowls.The juice of lemons makes fine punch.The box was thrown beside the parked truck.The hogs were fed chopped corn and garbage.Four hours of steady work faced us.Large size in stockings is hard to sell.

**Figure 3:**
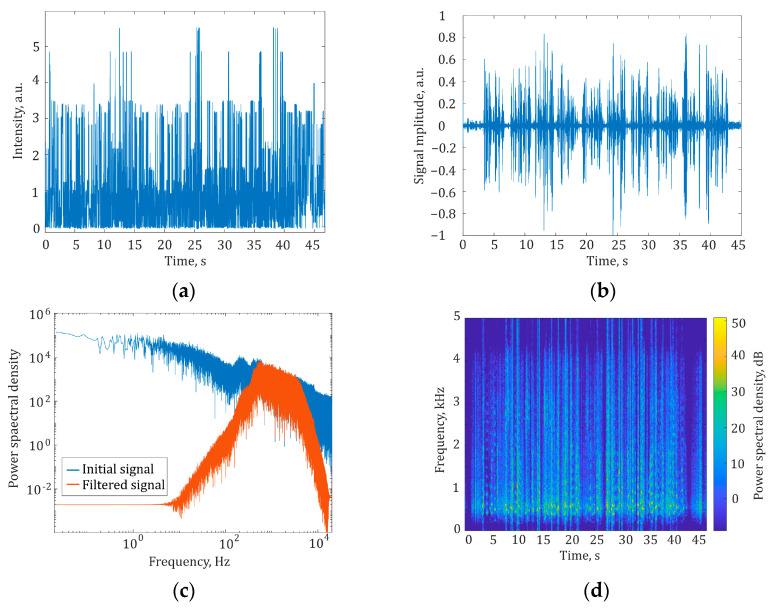
(**a**) Interference signal before preprocessing; (**b**) signal after preprocessing; (**c**) spectrum of simulated interference signal before (blue trace) and after (red trace) filtering; (**d**) the spectrogram of the speech signal after preprocessing.

Using the normalized shape of disturbance, *D*(*t*), coming from a recording of Harvard sentences as an acoustic speech disturbance acting on the sensing fiber of the φ-OTDR microphone, we obtained an output interference signal depending on various initial conditions while considering the system noises. Further, the received interference signal was processed according to the developed methodology presented in Section 2.3. The process of signal generation and obtaining a recognition-ready implementation is shown in the following figures. Figure 3a shows an interference signal in the form of intensity changes over time, simulated based on the developed model with a broad acoustic signal band of 40 kHz. Figure 3b shows the same signal after filtering with a bandpass filter in the frequency range from 500 Hz to 4 kHz to ensure effective audio filtering without losing useful information. The choice of the filtration frequency band will be further justified in Section 2.3. Figure 3c shows the spectrum of the simulated time-dependent interference signal before and after filtering, and Figure 3d shows the spectrogram of the filtered signal.

### 2.5. Speech-Recognition Technique in a Distributed Fiber-Optic Microphone Based on a Phase-Sensitive Optical Reflectometer

A distributed fiber-optic microphone is a device designed to register speech disturbances along the sensor fiber and recognize them, which distinguishes it from similar systems. To recognize words in the φ-OTDR signal, a speech recognition technique that includes a pre-processing algorithm and subsequent recognition is necessary [27].

**Figure 4:**
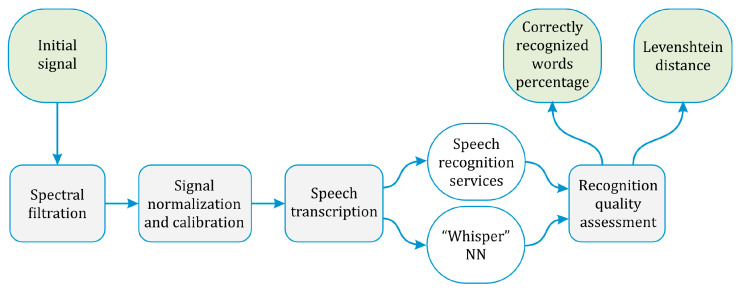
Block diagram of the algorithm used for processing the φ-OTDR setup signals.

For speech recognition, a novel processing and recognition technique has been developed for this work, which includes the following steps. Firstly, the source signal is filtered in the frequency range from 500 Hz to 4 kHz. This frequency band was chosen because the main contribution to the noise level is at frequencies below 500 Hz [13], while a negligible part of the sense-valuable speech signal is in a frequency range beneath 500 Hz. Conversely, frequencies above 4 kHz practically do not contribute to speech intelligibility [17], so we filter them out as well. After filtration, edge effects occur in the signal, which leads to an unnatural power increase within the time regions of [0…0.1*T*_meas_] and [0.9*T*_meas_…*T*_meas_]. Thus, secondarily, to avoid the edge effects of the filter, the filtered signal amplitude is normalized in the range from −1 to 1 and is multiplied by an empirically selected coefficient in a time region of [0.1*T*_meas_…0.9*T*_meas_]. Then, the received signal is converted to WAV format with the same sampling frequency as the original signal and recognized by both open speech-recognition services and neural networks. The block diagram of the algorithm is shown in Figure 4.

For speech recognition in the recorded audio WAV file, various recognition algorithms can be applied. For this purpose, we used five speech-recognition services, namely, “Yandex Translator” version 53.2, “Speechpad” version 9.9, “Google Documents” release 05/01/22, “Yandex SpeechKit” release 20/07/23, and the neural network “Whisper” version 2 [28]. These services accept the recorded WAV file and recognize words in this speech. The recognized speech can then be played and listened to by a user or used in the system to make decisions based on keywords. In our study, we compared recognized speech with original sentences in Listing 1 to provide a recognition quality assessment of the system.

Speech recognition quality is the main system characteristic of a distributed fiber-optic microphone based on an φ-OTDR. Therefore, it is important to evaluate this parameter objectively and quantitatively. We want to introduce and propose a new criterion for quantifying this parameter.

The most common quality estimation parameter is a signal-to-noise ratio, as shown in [11]. This criterion considers the overall ratio of signal power and noise over the entire signal duration. However, when low- and high-intensity signal segments appear one by one due to laser wavelength fluctuations, this estimation can become very unclear.

Since one is evaluating the speech signals as a sequence of a fixed number of words, it is appropriate to evaluate the quality by the percentage of correctly recognized words. This approach can be used to compare the sequence of words from each sentence obtained as a result of recognition with the original sentence. This is similar to the Bit Error Rate (BER) measurement and quality assessment parameters in telecommunication systems. The analysis of the correctly recognized word percentage shows us the degree of the transmitted text’s meaning preservation. For example, an error in just one letter (the word “blue” was obtained as a result of recognition instead of the word “glue”), which is likely to happen practically, can lead to a complete distortion of the semantic context of speech. However, such an assessment may be unnecessarily strict since, with this approach, replacing the word “glue” with any other, even absolutely unlike, e.g., “bloom,” introduces the same error as the word “blue.” Since the recognition algorithm can make mistakes when distinguishing between close phonemes and texts, in order to increase the informative value of the assessment and avoid its coarsening, it is also necessary to use a metric that allows quantifying the difference between two texts, character by character. It is advisable to achieve this by comparing each recognized sentence with the original. 

In our work, we also propose to use the Levenshtein distance [29] metric, which allows for the quantification of the difference between two sequences of characters. A sentence can be considered a sequence of characters, so when all the characters are registered correctly, the meaning of the sentence is complete. When there is no difference between any characters in the recognized sentence compared with the initial one, the Levenshtein distance is 0, which is the desired result. Therefore, in the worst speech recognition case, when the sentence is not recognized at all or is fully wrongly recognized, the Levenshtein distance value is equal to *M*, where *M* is the number of characters in the sentence. 

The Levenshtein distance metric was chosen becausee, unlike the Damerau–Levenstein distance [30], it does not consider the transposition of letters [31], which is a typical error when typing on the keyboard but not characteristic of speech and misunderstanding. The Levenshtein distance was chosen because other well-known metrics for evaluating the similarity of two strings are not suitable for the specific case of comparing recognized speech with a known reference phrase. For example, the Hamming distance [32] is not suitable since this algorithm works only with the same length of two strings, which, e.g., in the case of skipping one word, makes it impossible to assess the quality of recognition. When using the Jaro–Winkler distance [33,34], it is difficult to assess the similarity of two strings since the operations of deleting, inserting, and replacing characters are not considered. Also, it is difficult to find out how well the speech was recognized and how close the recognized sentence was to the reference one, which leads to a rougher estimate.

Thus, in our research, two approaches were chosen to assess the quality of speech recognition: the percentage of correctly recognized words, as the most intuitively obvious estimation, and the Levenshtein distance metric, as the most accurate and correct. These metrics allow an objective analysis of the speech recognition quality using a fiber-optic microphone based on an φ-OTDR.

## 3. Experiment

### 3.1. Description of Experimental Setups

**Figure 5:**
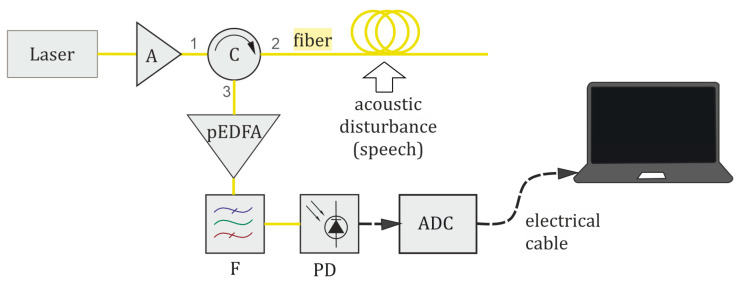
Generalized experimental setup.

To study the quality of speech recognition with a phase-sensitive reflectometer, a dedicated setup, shown in Figure 5, was created. Experimental studies of how the setup design parameters influence the quality of speech registration and recognition were carried out. The radiation of a continuous laser source is amplified by an amplifier A, if necessary. It goes to a circulator (C) that sends it along the path 1–2 to the sensing fiber with a length $L_{S}$, which can be formed differently. The far end of the sensing fiber is wound in several turns with a short radius of 4 mm, which is less than the minimum allowable for properly confined radiation guidance in our standard SM fiber. So, the laser radiation fades in such wound fiber turnings, and no intensity reflects from the far end of the sensor, which ensures the setup operates as a single section of the reflectometer. The different waves backscattered by the fiber refractive index inhomogeneities interfere with one another, and the interference signal travels to the optical preamplifier pEDFA through the circulator ports 2–3. Then, the optical filter F cuts off spontaneous emission pEDFA noise, and the photodetector PD detects the interference signal. The signal from the photodetector PD is sent to the ADC. The ADC sampling frequency *f_D_* can be varied if necessary. The signal is converted into digital form and then travels to a PC, on the screen of which one can see the interference signal in the form of a time-dependent intensity that changes when the fiber is affected by acoustic disturbances and speech. The setup represents a single “point” of a fiber line of a phase-sensitive reflectometer, i.e., it operates as a single fiber section within the spatial resolution determined by the pulse width according to the formula $l_{0}=\tau c/2n$, where *n* = 1.5 is the refractive index of the fiber. With a sensing fiber length of 20 m in the setup, we experimentally imitate data acquisition from one section of the phase-sensitive reflectometer with a probing pulse of duration τ = 200 ns (FWHM). At the output of the setup, one time sequence is recorded, which corresponds to a single cross-section of the waterfall reflectogram from one coordinate of the reflectometer line. Then, all further processing and recognition algorithms are applied to this time sequence.

Variation in a wide range of such reflectometer main parameters (such as the pulse repetition rate) is required in experiments to investigate the quality of speech recognition in different conditions. In a commercially available φ-OTDR, it is not feasible to quickly vary its parameters, which may require too much labor. To solve this problem, the special setup model of φ-OTDR single coordinate was developed. It does not require modulation of the radiation source intensity and allows using a lower-frequency ADC compared with a typical φ-OTDR. Thus, it becomes possible to quickly and easily vary the main parameters of a distributed microphone based on φ-OTDR by simply changing the parameters of the setup. The equivalent of the pulse width is changed by changing the length of the linear sensing fiber section. The equivalent of the pulse repetition rate is changed by a variation in the ADC sampling frequency. The signal-to-noise ratio at the output of the system can be varied by changing the configuration of the optical amplification circuit, i.e., by changing the parameters of the booster (A) and the preamplifier (pEDFA) combination, or by adding a variable attenuation to the optical signal. All the setup parameters and their correspondence to the φ-OTDR in the form of a ready-made device are given in Table 1.

**Figure 6:**
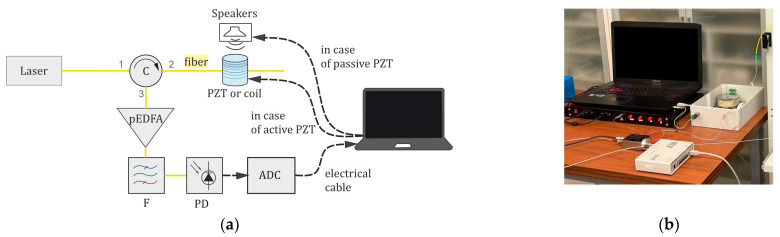
Experimental setup for the quality of speech recognition depending on the sampling frequency for hollow PZT cylinder with sensing fiber and coil sensing fiber configurations: (**a**) components and their interconnection in the experimental setup; (**b**) photo of the experimental setup.

In practice, the distributed microphone system must be able to register acoustic disturbances with quite different sound volumes. Many reasons may lead to a decrease in acoustic disturbance intensity, such as changes in distance from the source of an acoustic wave to the sensing fiber, changes in speech volume, and obstacles shielding sound propagation to the sensing fiber. Therefore, it is necessary to study the quality of speech recognition when fiber is perturbed by acoustic waves with different sound volumes. Various sensing fiber configurations were investigated as well. Thus, experimental studies were carried out in several stages:

1. A cylindrical PZT was used as a sensing element with a sensing fiber wound on it. The PZT was a hollow cylinder with an outer diameter of *D* = 85 mm, an inner diameter of *d* = 75 mm, and a height of *H =* 30 mm, with the length of the wound sensing fiber *L*_S_ = 20 m. The sensing fiber was influenced by the PZT vibrations, which occurred because of changes in the electrical signal applied to the PZT, which had a −3 dB bandwidth of Δ*f*_max_ = 16 kHz. The amplitude of the electrical signal applied to the PZT changed over time, according to an audio-recording of the Harvard sentences, so the sensing fiber wound on the PZT was stretched due to the PZT vibrations (an “active” PZT). A scheme and a photo of the experimental setup are shown in Figure 6a,b. 

At this stage of experiments, we verified the emulated results of the signal forming in a fiber-optical microphone based on a φ-OTDR. Acoustic disturbance applied to the fiber by the PZT is an idealized case when a realization of the sentences is clearly converted to fiber vibrations. Thus, it helped us conduct the first tests on how the ADC sampling frequency influences the quality of speech registration and recognition via the fiber-optical microphone.

2. A sensing fiber with a length of *L*_S_ = 20 m was wound into a passive coil with a diameter of 85 mm. Speakers playing recordings of Harvard sentences influenced the sensing fiber coil. 

Thus, this stage of experiments allowed us to conduct trials reproducing real conditions. Such a design of the sensing fiber allows us to maintain a high system sensitivity [35], since the speakers affect all of the fiber length. A scheme and a photo of the experimental setup are shown in Figure 6a,b. The same PZT, as in stage 1, was used as a coil, but the voltage was not applied to the PZT. The speakers playing the Harvard sentences audio recording were placed next to the “passive” PZT (without feeding any electrical signal to it).

**Figure 7:**
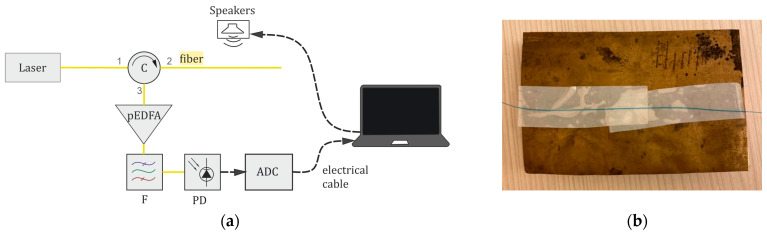
An experimental setup for experimental studies of the quality of speech recognition depending on the sampling frequency for the sensing fiber placed simply on a table: (**a**) an experimental setup circuit, (**b**) the use of a metal plate to increase the sensitivity of the system.

3. A straight sensing fiber with a length of *L*_S_ = 2.5 m was used, influenced by speakers playing recordings of Harvard sentences. The experimental setup scheme is shown in Figure 7a. This stage of experiments included several steps:

3.1. The fiber was placed simply on a table, without any auxiliary elements, to improve sound transmission to the optical fiber. 

3.2. The fiber was glued to a metal plate with dimensions of 80 × 50 × 0.2 mm^3^, as proposed in [12,36] and shown in Figure 7b. The metal plate was meant to increase the sensitivity of the system. It was mounted on legs so it could oscillate under the influence of an acoustic wave at the output of the speakers, transmitting vibrations to the optical fiber.

**Figure 8:**
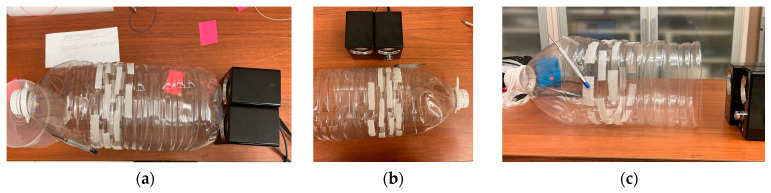
An experimental setup for studies of the quality of speech recognition depending on the sampling frequency using a sensing fiber with a length of 2.5 m wound around an elastic horn-like core, influenced by speakers with a sound volume of 72 dB(C): (**a**) with the bottle bottom influenced by the speakers; (**b**) with the bottle sidepiece influenced by the speakers; (**c**) with the horn-like bottle without a bottom influenced by speakers from inside.

3.3. The fiber was wound on an elastic core made of a plastic bottle, as proposed in [37], with a diameter of 140 mm. The bottle, as well as the metal plate in the previous experiment 3.2, made it possible to increase the amplitude of vibration transmission to the fiber. In this experiment, three regimes were studied: firstly, the original appearance of the bottle was kept, i.e., a bottle with a bottom, and speakers acted on it (Figure 8a); secondly, we cut off the bottom of the bottle, thus forming a horn, and the sidepiece was influenced by speakers (Figure 8b); and thirdly, the speakers were placed near the removed bottom and influenced the bottle from inside (Figure 8c). By changing the volume of the sound produced by the speakers, we investigated the behavior of such a system with different sound levels and various parameters.

**Figure 9:**
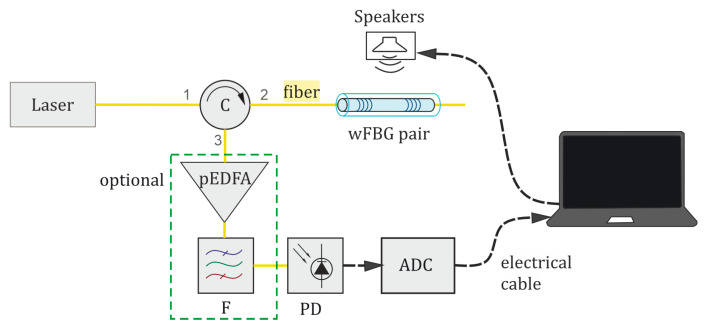
An experimental setup circuit for experimental studies of the quality of speech recognition depending on the sampling frequency for the sensing fiber with a pair of wFBGs.

4. A linear sensing fiber with a length of *L* = 2.5 m was used with a pair of wFBGs spaced by 1 m, as shown in Figure 9. Disturbance was applied in the middle of the fiber between the two wFBGs by speakers playing recordings of Harvard sentences. This configuration of the sensing fiber increases the intensity of the backscattered lightwaves, which increases the signal-to-noise ratio at the photodetector.

Since the length of the sensing fiber in all experiments was short (less than 1 km), there was no need to amplify the laser radiation with a booster. The pEDFA amplification varied depending on the configuration of the sensing fiber, as, in some cases, the intensity of the backscattered signal is sufficient to not need an optical preamplifier, but in other cases, it is not. Thus, we assembled two setups with or without a preamplifier and a filter to compare the results. 

The parameters of the setup in different experiments are given in Table 2.

The most important parameter to consider when designing a real system is the pulse repetition rate, because it defines the requirements for the ADC parameters, the latter of which is an expensive component of the system. However, most important is that the ADC sampling frequency *f_D_* physically limits the maximum sensing fiber length *L*_S_. The pulse repetition rate, in fact, is related to a maximum sensing fiber length as follows:

(15) $$f_{rep}=\frac{c}{2nL_{S}}$$

where n is the fiber effective refractive index, and c is light speed in a vacuum.

Table 3 shows how the maximum sensing fiber length relates to the pulse repetition rate of a φ-OTDR (the ADC sampling frequency of the experimental setup). Each section of a sensing fiber within a resolution length is equivalent to a one-point microphone placed with a resolution of *l*_0_ = 20 m. Thus, the equivalent number of one-point microphones in relation to the pulse repetition rate is shown in Table 3 as well.

A sensing fiber length of less than 10 km, such as *L*_S_ = 2.5 km, is sufficient for smart home technology implementations like the single fiber for multiple apartment concept and for other local applications in small areas and at short distances. This will help with the development of advanced devices. However, when it is necessary to cover larger distances for some applications, such as integration in a fiber communication line using a comprehensive remote monitoring system [38] or distributed monitoring of roads [39] in smart city infrastructure with the function of speech recognition, a sensor length *L*_S_ of tens of kilometers must be ensured. At the moment, long-distance DAS are most in demand. Therefore, the issue of ensuring high recognition quality at sampling frequencies of less than 10 kHz remains quite important, and this problem can be solved by increasing the system sensitivity [37].

Other φ-OTDR parameter variations, such as adjusting the optical amplification required in practice, can be easily achieved by changing the pEDFA gain. The pulse duration can be changed easily as well by simply adjusting AOM parameters. Therefore, we paid the most attention to how the ADC sampling frequency in our setup influences the quality of speech recognition.

In each experiment, the dependence of the quality of speech recognition on the ADC sampling frequency was studied using five speech-recognition services. Each service was used for speech recognition with different sampling frequencies of 3, 5, 10, 20, and 40 kHz. For each sampling frequency, the percentage of words correctly recognized was calculated, thus making possible the intercomparison of these services by quality numbers. This procedure allowed us to choose the services that recognize speech best. For the recognition services chosen, the quality of speech recording with our microphone and speech recognition were more precisely studied. As a result, curves of the percentage of recognized words and the Levenshtein distance with dependence on the ADC sampling frequency were obtained, which are equivalent to the dependence on the pulse repetition rate in a φ-OTDR. After analyzing the obtained dependencies, the most effective sampling frequencies were selected to study the influence of other system parameters on the recognition quality. For example, when the fiber was affected by the sound from the speakers, a study of the recognition quality dependence on the sound volume measured near the sensing fiber was conducted.

**Figure 10:**
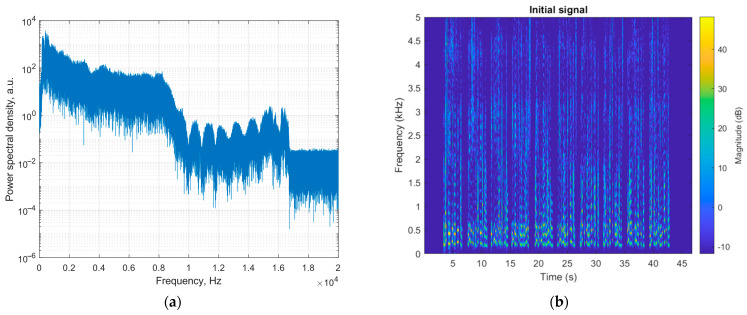
Spectral characteristics of the original audio recording: (**a**) Spectrum; (**b**) Spectrogram.

The analysis of signal spectrograms is also informative. It allows us to evaluate the frequency composition of the signals received and thus determine why the particular configurations of sensing fiber provide good or bad speech recognition in a distributed fiber microphone based on φ-OTDR. Figure 10a,b shows the spectrum and a spectrogram of the audio recording applied to the sensing fiber in experiments. The spectrum (Figure 10a) is calculated as the FFT from the original audio recording, where the 10 Harvard sentences from Listing 1 follow one another with 1 s spacing. As one can see, the spectrum shows which spectral components have ever existed in the realization, but the moments of their presence are unknown. This is a drawback of estimating the spectral composition using the spectrum and why the spectrogram (Figure 10b) is needed to properly conduct a comparative analysis of the obtained spectral composition with an initial one. The original audio recording was obtained with a sampling frequency of 44 kHz, while the spectrogram is presented in the frequency range up to 5 kHz for better visualization since spectral components with frequencies above 5 kHz have a low magnitude and do not make a significant contribution to the spectrum but rather increase the noise. The spectrogram shows the frequency composition as a function of time for each sentence. The sentences can be easily distinguished, as there are 10 regions in the 3D plot, separated in time by dark-blue silence regions (of 1 s duration) with a magnitude of less than −10 dB (“silence”). Subsequently, we compared all the newly obtained and reconstructed spectrograms with this original 3D plot. The experimental results obtained in different experiments and a discussion are given further.

### 3.2. Results of Experimental Studies of Quality of Speech Recognition with Different Services for Different Sensing Fiber Configurations 

Table 4 and Table 5 show how the percentage of correctly recognized words depends on some ADC sampling frequencies in a distributed fiber-optic microphone based on φ-OTDR with a sensing fiber wound around a hollow PZT cylinder and a coil sensing fiber. The time sequences in the setup’s output, shown in Figure 6, were gathered and recognized with the five services in both cases.

Table 6 shows the results of speech recognition using Yandex SpeechKit (YS) and Whisper NN for sampling frequencies of 20 kHz and 40 kHz and different sound volumes of the acoustic wave from the speakers acting on the fiber coil. The sound volume was measured by a smartphone with a sound meter app near the coil perpendicular to the speakers. We selected three values of sound volume for the PC-controlled audio recording, which are given in Table 6. The sound volumes in the experiments, which ranged from 70 to 92 dB(C), are quite high and lie within the range of loud conversations.

In Table 7, the experimental results of speech recognition depending on the ADC sampling frequency are given for a straight sensing fiber in two cases: when the fiber is placed simply on a table (stage 3.1) and when the fiber section with a 0.8 m length is glued to a metal plate (stage 3.2). We used Yandex SpeechKit and Whisper NN for speech recognition of gathered signals, as in previous experiments. The average sound volume measured near the sensing fiber was 89 dB(C). Studies at lower volume levels have also been conducted, but in these cases, the quality of speech recognition was reduced greatly, similar to experiments with a coiled sensing fiber, and these results are uninformative. 

Table 8 shows the results of speech recognition in signals gathered with an ADC sampling frequency of 40 kHz while varying the volumes of the Harvard sentences audio record produced by the speakers and using a sensing fiber wound around an elastic core. The sound volumes given in Table 8 were measured at the output of the speakers installed near the bottle.

For the fiber microphone based on a φ-OTDR with wFBGs, the quality of speech signal recognition was investigated for a sound volume of 108 dB(C). Table 9 and Table 10 show the results for Yandex SpeechKit and Whisper NN, since other algorithms did not recognize speech with adequate quality when a sensing fiber with a pair of wFBGs was used. 

**Figure 11:**
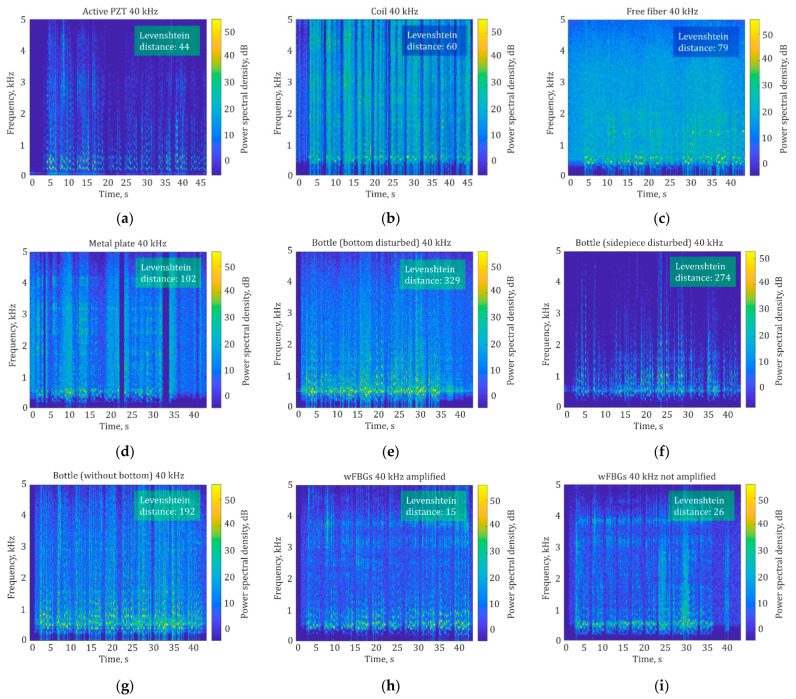
Spectrograms of signals obtained with a sampling frequency of 40 kHz: (**a**) a PZT-actuated disturbance; (**b**) a coiled sensing fiber, with a volume of 92 dB(C); (**c**) a sensing fiber placed simply on the table, with a volume of 89 dB(C); (**d**) a sensing fiber section 0.8 m long glued to a metal plate, with a volume of 89 dB(C); (**e**) a bottle bottom influenced by the speakers, with a volume of 72 dB(C); (**f**) a bottle sidepiece influenced by the speakers, with a volume of 72 dB(C); (**g**) a horn-like bottle without a bottom influenced by the speakers from inside, with a volume of 72 dB(C); (**h**) a sensing fiber with wFBGs 1 m apart with a preamplifier, influenced by speakers, with a volume of 108 dB(C); (**i**) a sensing fiber with wFBGs 1 m part without a preamplifier, influenced by speakers, with a volume of 108 dB(C).

Figure 11 shows spectrograms of signals gathered with a sampling frequency of 40 kHz for different types of disturbance and sensing fiber configurations.

The spectrograms are built using a sliding Hanning window with a length of *H*_SP_ = 2000 samples. Each spectrogram is marked with the Levenshtein distance value calculated after speech recognition of the corresponding signal.

As shown in Table 4 and Table 5, the best recognition quality is provided by Yandex SpeechKit and Whisper NN when the ADC sampling frequency is 40 kHz. Other recognition services (Yandex Translate, Speechpad, and Google Documents) do not provide good speech recognition, so the percentage of correctly recognized words is zero or very low for a sensing fiber wound around both “active” and “passive” PZT. At sampling frequencies of 3 and 5 kHz, low recognition quality for all five services is caused by bad speech recording with the microphone because it cuts off high frequencies via low *f*_D_. Meanwhile, at high frequencies from 10 to 40 kHz, Yandex SpeechKit and Whisper NN give better results, by 50% to 70%, compared with Yandex Translate, Speechpad, and Google Documents, which might be caused by the better-developed recognition algorithms of these services. In the spectrogram of a signal gathered with “active” PZT disturbance and a sampling frequency of 40 kHz, the sentences can be clearly distinguished from each other, and the spectrogram in Figure 11a is quite similar to the initial one in Figure 10b. 

For the coiled sensing fiber, the most intense acoustic signal with a volume of 92 dB(C) is recognized best, as shown in Table 6. As expected, the lower the intensity of the acoustic disturbance, the lower the percentage of correctly recognized words. When decreasing the sound volume from 92 dB(C) to 80 dB(C), the percentage of correctly recognized words drops by half using Whisper NN. When the volume is at the typical conversation level, i.e., 50 dB(C), speech cannot be recognized. The spectrogram for the coiled sensing fiber is more noisy. It has high-power components (up to 50 dB in magnitude, shown in Figure 11b in yellow) in a wide frequency range above 5 kHz, unlike the original one, so it is difficult to distinguish the wanted frequencies from high background noise. We assume this is due to the much higher sensitivity of the 20 m fiber coil, which has approximately 75 turns with a winding diameter of 85 mm. However, to confirm this, it is necessary to exclude the possibility of signal wrapping at a high sound volume, which may also cause high noise in a spectrum.

Table 7 shows that, for a pulse repetition rate above 10 kHz, high-quality speech recognition of up to 75% can be achieved, even with a sensing fiber placed simply on the table. The spectrogram in Figure 11c shows that the 10 sentences can be distinguished from each other (in the frequency range of 0.5–2 kHz, there are regions of higher magnitude), but it is very noisy. However, the frequency of 10 kHz is quite high and limits the parameters of a φ-OTDR, according to Table 3, and thus *L*_S,max_ = 10 km. For a sampling frequency lower than 10 kHz, a metal plate should be used to ensure more efficient sound conversion into fiber vibrations. In the case of using a metal plate at sampling frequencies higher than 10 kHz, there is a huge drawback, not typical of any other cases. In the spectrogram in Figure 11d for 40 kHz, some dead zones appear during the 22nd and 33rd seconds (which are displayed as dark-blue regions). During these time intervals, the fiber does not vibrate, and the speaker’s sound is not registered. We associate such artifacts with the natural frequencies of the plate, which leads to distortions and degrades the desired signal. Thus, we can conclude that using a metal plate for sound transmission enhancement leads to many limitations.

Table 8 shows that influencing the bottom of the bottle and the bottle sidepiece, unlike other sensing fiber configurations, yields a bad recognition quality of less than 15% of correctly recognized words, even at a high sound volume of 92 dB(C), which is caused by the registered signal deteriorations and can be explained as follows: When the bottom of the bottle is perturbed, it vibrates intensively, which increases both wanted acoustic waves from the speakers and unwanted ones coming from the environment. The mix of waves undergoes multiple reflections in an internal cavity of the bottle, which leads to a significant background level and parasitic wave emergence. The signal-to-noise ratio decreases because of unwanted acoustic-wave multiple amplifications. One can observe this effect in the spectrogram shown in Figure 11e as an increase in the noise level, so it is impossible to distinguish the sentences from each other. When the bottom and sidepiece (Figure 11f) of the bottle are influenced, the most significant spectral components in the spectrograms are in a frequency range of about 0.5 kHz and 1 kHz, probably due to the natural frequencies of the bottle with a bottom. Perturbing the horn-like bottle from the inside yields good speech recognition quality at the lowest volume level of 72 dB(C), so in the spectrogram (Figure 11g), the sentences can be distinguished separately from each other, the speech is transmitted without significant distortion, and the noise level is quite low. Visually, the spectrogram is similar to the numerical simulation result based on the developed mathematical model. 

Analyzing the results of speech recognition quality using the wFBGs given in Table 9 and Table 10, one can see that such a configuration of a sensing fiber yields the highest quality of speech recognition, which is also demonstrated in the spectrograms for a sampling frequency of 40 kHz in Figure 11h (without a preamplifier, the percentage is 96% and the Levenshtein distance is 15) and in Figure 11i (with a preamplifier, the percentage is almost 94% and the Levenshtein distance is 26), that are similar to the spectrogram of the initial signal, and on each of them the boundaries between the sentences can be clearly distinguished separately. However, in Figure 11i, unstable sensitivity regions are observed at the beginning of sentences six and eight, which are in an increased-sensitivity region. This may be due to random changes in the propagating light polarization state, which leads to the polarization-induced fading (PIF) effect [40]. If a preamplifier is used, some parasitic high-power spectrum components appear in a frequency region of 3.5 kHz, but they do not affect the quality of speech recognition dramatically. As shown in Table 9 and Table 10, the sensing fiber with wFBGs allows word recognition even at low sampling frequencies of 5 and 10 kHz. Thus, artificial reflectors such as wFBGs in the sensing fiber yield quite a stable signal, which is a promising result.

### 3.3. Quality of Speech Recognition Competitive Analysis for Different Sensing Fiber Configurations

**Figure 12:**
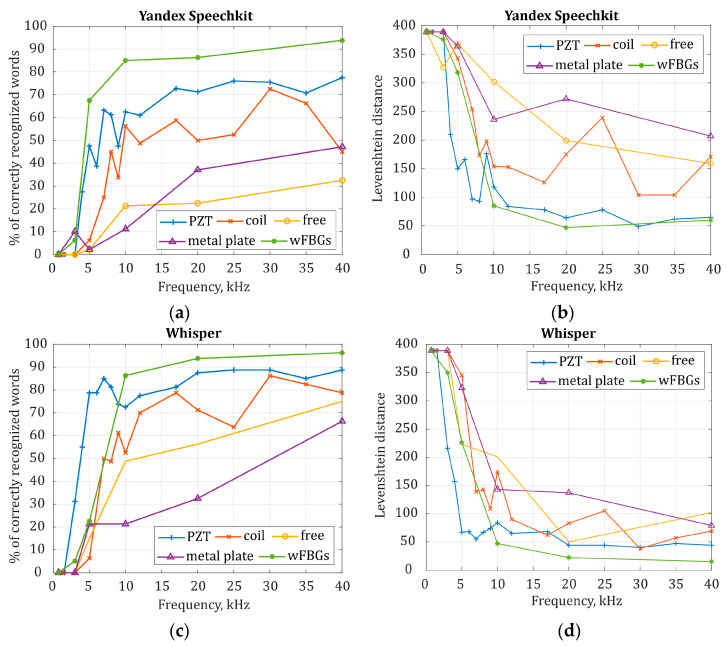
Dependence of speech recognition quality on ADC sampling frequency for different sensing fiber configurations: (**a**) the percentage of words recognized by Yandex SpeechKit; (**b**) the Levenshtein distance of the words recognized with Yandex SpeechKit; (**c**) the percentage of words recognized by Whisper NN; and (**d**) the Levenshtein distance of the words recognized with Whisper NN.

For Yandex SpeechKit and Whisper NN, which generally demonstrated the best results in speech recognition quality, curves of the percentage of recognized words and the Levenshtein distance were obtained depending on the ADC sampling frequency in a range from 500 Hz to 40 kHz, as presented in Figure 12a–d.

The result of speech recognition at different frequencies in the experimental setup using the PZT with a sensing fiber is shown in Figure 12 in blue. When the ADC sampling frequency *f_D_* is less than 5 kHz, the quality of speech recognition decreases significantly. The percentage of recognized words and the Levenshtein distance tend to be 0 and 386 (when *M* = 386 is the number of characters in Harvard sentences Listing 1), respectively, which means that speech is not recognized at all. With an increase in the sampling frequency and, consequently, with an increase in the pulse repetition rate, the quality of speech recognition improves, which is assessed by both metrics. The percentage of recognized words increases and reaches 77.5% and 88.75% for the Yandex SpeechKit and the Whisper NN, respectively, and the Levenshtein distance decreases to 50 when the sampling frequency reaches 40 kHz. One can identify a certain cut-off sampling frequency, i.e., a threshold sampling frequency, above which high-quality recognition is ensured, the percentage of recognized words is above 70%, and the Levenshtein distance is less than 100. For the Yandex SpeechKit, the cut-off ADC sampling frequency is 15 kHz, and for the Whisper NN, it is 5 kHz. When the ADC sampling frequency *f_D_* is greater than 20 kHz, the recognition quality increases insignificantly, and a kind of saturation appears. The nonlinear nature of the plot might be due to a random distribution of light amplitude and phase wrapping, which affects the signal-to-noise ratio at the PD. It is interesting that for the Whisper NN, the quality of recognition does not grow proportionally to the sampling frequency increase but instead has a threshold nature. At sampling frequencies below 5 kHz, speech is not recognized at all, and at a frequency of 5 kHz and higher, speech is recognized with high quality up to 70% (the Levenshtein distance of 55). The results of these experiments allow us to conclude that the pulse repetition rate of φ-OTDR significantly affects the quality of the recorded interference signal, which determines the subsequent quality of speech recognition. Thus, the quality of signal registration with fiber, and hence the quality of speech recognition proportionally, depends on the pulse repetition rate of the φ-OTDR. Below the cut-off sampling frequency, all the words are badly transmitted because they lose information contained in high speech frequencies. Hence, recognition services are not able to recover words from the sounds. Further, the higher the pulse repetition rate of the φ-OTDR, the better the speech recognition quality. However, after reaching a limitation (typical for a specific sensing fiber configuration), the recognition quality does not grow further when increasing the pulse repetition rate because of the increasing noise as well as the wanted signal at higher frequencies.

It is also important to compare experimental results when a coiled sensing fiber or a sensing fiber with a length of 2.5 m simply placed on the table is perturbed by the speakers. For the coiled sensing fiber, the speech recognition quality curve is shown in Figure 12 in red. It is more rugged compared with the one obtained with PZT disturbance, which is probably due to the peculiarities of sound wave propagation from the speakers along the sensing fiber. However, for the Whisper NN, a cut-off recognition frequency is about 10 kHz. At an ADC sampling frequency of 25 kHz, there is a sharp deterioration of the recognition quality, perhaps because of some random ambient acoustic noise increasing during this experiment. However, for higher sampling frequencies, the quality of recognition is better, and the percentage of words recognized reaches 72.2% (the Levenshtein distance is 104). At a sampling frequency of 40 kHz, the recognition quality decreases again. This characterizes the microphone with a coiled sensing element as unstable over time, probably because of its high sensitivity to both wanted (speech) and unwanted (acoustic noise) disturbances.

As per the setup with a sensing element glued to a metal plate (the curve is shown in purple in Figure 12), for some sampling frequencies, the recognition quality also drops sharply. However, in general, the quality of speech recognition increases with the sampling frequency. Such a sensing fiber configuration allows speech recording and recognition at frequencies lower than 10 kHz, better than the others, except for the sensing fiber with wFBGs. When comparing the three dependencies obtained with the Whisper NN for a coiled sensing fiber, a sensing fiber placed simply on the table (yellow curve), and a sensing fiber glued to a metal plate, one can see that at a sampling frequency of about 5 kHz, the best result is obtained with a sensing fiber glued to a metal plate. However, when the sampling frequency is greater than 10 kHz, the quality of speech recognition using a coiled sensing fiber becomes higher than the other two configurations. Thus, for a pulse repetition rate of more than 10 kHz, increasing sensitivity using a metal plate becomes unsuitable since, in this case, the result is the worst: the speech recognition quality is less than 66.3% (the Levenshtein distance is 102).

The results demonstrate that it is fundamentally possible to recognize speech recorded by a fiber microphone based on φ-OTDR with a pulse repetition rate of *f*_rep_ > 10 kHz with an accuracy of more than 50%, even when a linear sensing fiber is simply placed on a table. If there is an issue of increasing the quality of speech recognition without increasing the pulse repetition rate, a coiled fiber can be used, which can increase the system’s sensitivity and, in practice, be easily implemented in the case of short-length paths. The best recognition quality, when a real acoustic disturbance is applied to the fiber by the speakers, is obtained when a sensing fiber with wFBGs is used. The results obtained with a wFBG sensing fiber and those obtained when a hollow PZT cylinder with a sensing fiber was used are interesting to compare in two spectral ranges and using two recognition methods. For the Whisper NN, the cut-off recognition frequency with the PZT actuator is 5 kHz, and in a frequency range less than 10 kHz, the speech recognition quality in a scheme with the sensing fiber wound around the PZT is the best. At the same time, for a sensing fiber with wFBGs, the cut-off recognition frequency is 10 kHz, and when *f_D_* = 3 kHz and *f_D_* = 5 kHz, few words are recognized by the Whisper NN, so the percentage of recognition is 30%, and the Levenshtein distance is more than 200. Thus, when the fiber is perturbed by the speakers, wFBGs make it possible to ensure the best recognition quality and stable operation of the fiber microphone. At a lower pulse repetition rate, recognition quality significantly depends on the recognition algorithm. As seen in the experimental results, it is clear that the Yandex Speechkit recognizes almost 70% of words at *f_D_* = 5 kHz, while the Whisper NN recognizes less than 30%.

An important result is that the speech recognition quality increases with the pulse repetition rate, which limits the spectrum that can be recorded. A low pulse repetition rate results in a loss of high-frequency components. High frequencies in human speech are important, as they carry meaning. They are not only used to express emotions and intonation but also to help distinguish sounds and words in a speech, give brightness and clarity to the voice, and improve speech intelligibility in noisy environments. Therefore, even using effective neural network algorithms, recognition quality above 75% is ensured only at high pulse repetition rates. However, this limits the maximum length of the sensing fiber. Thus, it is necessary to find a compromise between the available sensing fiber length and the desired quality of speech recognition, which depends on the task specification to be solved. For example, when increasing the pulse repetition rate, only a shorter sensing fiber length is possible, which allows for obtaining a higher quality of speech recognition within the observed area. This might be preferable when it is necessary to clearly capture all commands with an error of less than a few words, for example, for voice control systems within a limited area of one apartment, office, etc. When one needs to increase the length of the sensor, only a lower recognition quality can be obtained, but this allows, for example, serving more users simultaneously without increasing equipment costs. In this case, one can recognize only the keywords of the conversation, which, for example, will mark that at a specific coordinate along the sensor, there is a request for some service, and subsequently, the command can be clarified when contacting again. 

## 4. Conclusions

In this study, a novel approach to a fiber microphone for speech recognition based on a φ-OTDR prototyping is proposed. Using the developed mathematical model of a distributed fiber microphone based on an φ-OTDR, a theoretical study of the system parameters influencing the quality of speech recognition was conducted, and the main reasons for recognition quality deterioration, such as system noise and external unwanted influences, were determined. The proposed configurations of the sensing fiber helped in the investigation of the quality of the recognition’s dependence on its sensitivity to speech. The developed recognition technique, using open-source services for speech recognition and an open neural network, enabled us to analyze the quality of speech recognition using different algorithms and compare them. Experimental studies of the speech recognition quality for different pulse repetition rates from 0.5 to 40 kHz and different speech sound volumes were conducted. More than 90% of correctly recognized words in a standard phonetically balanced phrase, in the form of Harvard sentences containing 80 words, were obtained with a pulse repetition rate *f*_rep_ > 10 kHz, while with *f*_rep_ < 10 kHz, the percentage was no greater than 70%, which nevertheless is also a good and technically relevant achievement. As a result, a sensing fiber with wFBGs is the best configuration for a distributed fiber microphone based on φ-OTDR, ensuring effective speech recording and recognition quality up to 96% (a Levenshtein distance of 15) at a sampling frequency of 40 kHz.

In general, the results presented demonstrate that a fiber-optic microphone based on φ-OTDR has a high potential for use in distributed speech recording and recognition systems, but a high-precision speech-recognition algorithm should be applied. We propose this to be the “Whisper” NN or the “Yandex Speechkit” service. However, other researchers and users may test other recognition algorithms, including those of their own development. Even a simple φ-OTDR fiber sensor, without phase recovery, ensures speech registration and high-quality recognition with various sensor configurations. This allows us to propose such a system for use in the smart-home or smart-city concept and other applications. Experience from previous developments and the work demonstrated prove the technical feasibility of implementing a distributed fiber microphone with speech recognition based on φ-OTDR. Further improvements in the recognition algorithms will allow such systems to be taken to a new level, opening the possibility for a wide implementation of such fiber-optical acoustic sensors with speech recognition in everyday life.

## Figures and Tables

**Table 1:** Correspondence of the setup parameters to the distributed microphone based on φ-OTDR in the form of ready-made device parameters.

Parameters of an φ-OTDR to be Investigated	Parameters of the Experimental Setup to be Changed
Resolution, *l*_0_	Length of the sensing fiber, *L*_S_
Pulse repetition rate, *f*_rep_	ADC sampling frequency, *f_D_*
Signal-to-noise ratio at the photodetecor	Booster or preamplifier gain + attenuation

**Table 2:** Experimental setup parameter summary.

No.		Sensing Fiber Configuration	Disturbance Type	pEDFA Output Power, dBm
1		A hollow PZT cylinder with an outer diameter of *D* = 85 mm, an inner diameter of *d* = 75 mm, and a height of *H =* 30 mm, with *L*_S_ = 20 m of fiber wound on it	An electrical signal on the PZT with an amplitude varying according to the Harvard sentences recording	0
2		A fiber with length of *L* = 20 m, wound into a coil with a diameter of 85 mm	Recording of Harvard sentences played by speakers	4
3		A fiber with length of *L*_S_ = 2.5 m	Recording of Harvard sentences played by speakers	6
	3.1	placed simply on a table		
	3.2	A fiber with length of *L*_S_ = 2.5 m glued to a metal plate with dimensions of 80 × 50 × 0.2 mm^3^		
	3.3	A fiber with length of *L* = 2.5 m wound on a plastic bottle with a diameter of 140 mm		
4		A fiber with length *L*_S_ = 2.5 m with a pair of wFBGs spaced by 1 m	Recording of Harvard sentences played by speakers	−15

**Table 3:** The main φ-OTDR’s parameters relation to the pulse repetition rate.

Pulse Repetition Rate, *f*_rep_, kHz	Maximum Sensing Fiber Length, *L*_S_, km	Equivalent Number of One-Point Microphones
1	100	5000
5	20	1000
10	10	500
40	2.5	125

**Table 4:** The quality of speech recognition with five algorithms for a setup using the “active” PZT with a sensing fiber.

Sampling Frequency, kHz	% of Recognized Words				
	Yandex Translate	Speechpad	Google Documents	Yandex SpeechKit	Whisper NN
3	0.0	0.0	0.0	0.0	28.1
5	2.5	1.3	0.0	48.1	70.8
10	2.5	3.8	3.8	63.3	65.2
20	29.1	5.1	5.1	72.2	78.7
40	48.1	36.7	54.4	78.5	79.8

**Table 5:** The quality of speech recognition with five algorithms for a setup using coiled sensing fiber on a passive PZT cylinder influenced by speakers.

Sampling Frequency, kHz	% of Recognized Words				
	Yandex Translate	Speechpad	Google Documents	Yandex SpeechKit	Whisper NN
3	0.0	0.0	0.0	0.0	0.0
5	0.0	0.0	0.0	3.4	5.6
10	0.0	1.1	0.0	50.6	47.2
20	2.2	0.0	0.0	44.9	64.0
40	2.2	0.0	1.1	40.4	70.8

**Table 6:** Quality of speech recognition by Yandex SpeechKit and Whisper NN for sampling frequencies of 20 kHz and 40 kHz and different sound volumes of the acoustic wave perturbing the coiled sensing fiber.

Sound Volume, dB(C)	% of Recognized Words			
	YS		Whisper NN	
	20 kHz	40 kHz	20 kHz	40 kHz
72 dB(C) average	0.0	0.0	0.0	0.0
80 dB(C) average	2.5	5.0	35.0	40.0
92 dB(C) average	26.3	47.5	61.3	71.3

**Table 7:** Quality of speech recognition with different services when a sensing fiber was placed simply on the table and when a sensing fiber was glued to a metal plate.

Sampling Frequency, kHz	% of Recognized Words			
	A Sensing Fiber on the Table		A Sensing Fiber Glued to a Metal Plate	
	YS	Whisper NN	YS	Whisper NN
3	0.0	0.0	10.1	0.0
5	2.2	15.0	2.2	21.3
10	21.3	48.8	11.2	21.3
20	22.5	56.3	37.1	32.5
40	32.5	75.0	47.2	66.3

**Table 8:** Quality of speech recognition with different services when a sensing fiber was wound at an elastic horn-like core for a sampling frequency of 40 kHz.

Sound Volume, dB(C)	% of Recognized Words					
	The Bottle Bottom		The Bottle Sidepiece		The Bottle without a Bottom	
	YS	Whisper	YS	Whisper	YS	Whisper
92 dB(C)	10.0	11.3	0	0	0	0
80 dB(C)	6.3	13.8	6.3	12.5	0	0
72 dB(C)	3.8	11.0	5.0	11.3	16.3	36.3
65 dB(C)	0	0	0	0	0	0

**Table 9:** Five algorithms’ percentage of speech recognized for a setup using a sensing fiber with a pair of wFBGs.

Sampling Frequency, kHz	% of Recognized Words			
	YS		Whisper NN	
	No Amplification	Amplified	No Amplification	Amplified
3	3.7	6.3	0	5.0
5	22.7	67.5	7.7	22.5
10	62.0	85.0	70.8	86.3
20	59.4	86.3	82.2	93.8
40	81.0	93.8	79.7	96.3

**Table 10:** Levenshtein distance of speech recognition for five algorithms in a setup using a sensing fiber with a pair of wFBGs.

Sampling Frequency, kHz	Levenshtein Distance			
	Yandex SpeechKit		Whisper NN	
	No Amplification	Amplified	No Amplification	Amplified
3	368	376	286	350
5	286	318	133	226
10	105	85	55	47
20	133	47	48	22
40	44	60	26	15

## Data Availability

The data presented in this study are available on request from the corresponding author.
